# Royal Jelly as a Natural Endocrine Modulator of Serum Estradiol Levels in Juvenile Sterlets (*Acipenser ruthenus*)

**DOI:** 10.3390/molecules31071210

**Published:** 2026-04-06

**Authors:** Dragoș Moraru, Ersilia Alexa, Adrian Grozea, Violeta Igna, Sandra Antonia Mihailov, Christine Neagu, Silvia Pătruică

**Affiliations:** 1 Faculty of Bioengineering of Animal Resources, University of Life Sciences “King Mihai I” from Timișoara, Calea Aradului No. 119, 300645 Timișoara, Romania; dragos.moraru@usvt.ro (D.M.); sandramihailov@usvt.ro (S.A.M.); silviapatruica@usvt.ro (S.P.); 2 Faculty of Food Engineering, University of Life Sciences “King Mihai I” from Timișoara, Calea Aradului No. 119, 300645 Timişoara, Romania; christine.neagu@usvt.ro; 3 “Food Science” Research Center, University of Life Sciences “King Mihai I” from Timişoara, Aradului Street No. 119, 300645 Timişoara, Romania; 4 Faculty of Veterinary Medicine, University of Life Sciences “King Mihai I” from Timișoara, Calea Aradului No. 119, 300645 Timişoara, Romania; violetaigna@usvt.ro

**Keywords:** royal jelly, endocrine modulator, steroid hormones, HPLC analysis

## Abstract

The present study investigates the role of royal jelly as a natural endocrine modulator of serum estradiol levels in juvenile sterlets (*Acipenser ruthenus*), a species of major interest for sustainable aquaculture. The experiment was conducted over a period of 85 days under controlled recirculating system conditions, using four dietary treatments (*n* = 30 fish per group): a control group and three groups supplemented with 1%, 3%, and 5% royal jelly. Serum estradiol concentrations were determined by high-performance liquid chromatography (HPLC), while biometric assessment included the determination of total length (L), standard length (Sl), maximum body height (H), body circumference (C), and body mass (BM). Royal jelly supplementation significantly increased serum estradiol levels in a dose-dependent manner (*p* < 0.05), with the highest values recorded in the 5% group compared to the control. The proportion of individuals with non-detectable estradiol levels decreased progressively with increasing supplementation level. Biometric analysis revealed moderate effects on growth parameters, with no statistically significant differences among groups for most traits (*p* > 0.05), except for maximum body height, which showed a significant overall effect (ANOVA, *p* = 0.0089). Principal Component Analysis (PCA) highlighted the relative independence between endocrine variability and growth dynamics. Overall, the findings support the potential of royal jelly as a natural endocrine modulator of serum estradiol, representing a promising and environmentally friendly alternative to synthetic hormonal substances used in aquaculture. This approach may contribute to the development of innovative nutritional strategies for endocrine control and the optimization of biological performance in sturgeons, in accordance with the principles of sustainable aquaculture.

## 1. Introduction

In the context of modern aquaculture development, there is increasing interest in identifying natural alternatives to synthetic additives used in reproductive technologies, with the aim of improving production performance while enhancing health status and physiological balance and simultaneously reducing environmental impact [1]. Functional feeds enriched with bioactive compounds of natural origin represent a promising strategy for optimizing fish production and supporting sustainable aquaculture practices [2,3].

Royal jelly is a natural secretion produced by worker bees (*Apis mellifera*) for the nourishment of larvae and the queen, and is recognized for its complex composition and high biological value [4]. It contains high-quality proteins, essential amino acids, bioactive lipids, B-complex vitamins [5], minerals, and numerous biologically active compounds [6], including substances with antioxidant [7], immunomodulatory, and endocrine-modulating potential [8]. Owing to these properties, royal jelly has been extensively investigated in animal nutrition, demonstrating beneficial effects on metabolism, growth performance, and endocrine and reproductive functions [9].

In recent years, interest in the application of royal jelly in aquaculture has increased considerably. Available studies suggest that dietary supplementation with this natural product may enhance feed utilization efficiency, growth rate, and physiological responses in fish [10]. However, data regarding the influence of royal jelly on endocrine function in aquaculture species remain limited, particularly in sturgeons [11].

Sturgeons represent a group of fish with significant economic and ecological value, being important both for meat and caviar production and for conservation programs. The sterlet (*Acipenser ruthenus*) is characterized by a relatively rapid growth rate, good adaptability to controlled rearing conditions, and early sexual maturation, making it a species of major interest for European aquaculture [12]. The optimization of nutritional strategies for this species is essential not only for improving production efficiency but also for supporting sustainable aquaculture practices.

Moreover, nutritional interventions applied during early developmental stages may influence endocrine processes, including steroidogenesis and sexual differentiation. The use of natural compounds capable of modulating the androgen–estrogen hormonal balance represents an attractive alternative to synthetic hormonal substances, generating both scientific and applied interest [13]. However, the mechanisms through which royal jelly may influence the steroid profile in sturgeons remain insufficiently investigated. Its use as a natural endocrine modulator of serum estradiol levels in juvenile sterlets (*Acipenser ruthenus*) could provide a valuable direction for future research on sexual differentiation. It is worth noting that caviar is obtained exclusively from female sterlet; therefore, experimental groups exhibiting elevated estradiol levels may suggest an increased proportion of females, which would have major economic implications for caviar production.

The novelty of the present study resides in the integrated evaluation of royal jelly as a natural endocrine modulator in juvenile sterlets (*Acipenser ruthenus*), combining precise chromatographic quantification of serum 17β-estradiol by HPLC with a comprehensive biometric assessment and multivariate statistical analysis. Although royal jelly has been previously investigated in fish nutrition, data regarding its direct influence on steroid hormonal profiles in sturgeons remain scarce, and studies addressing endocrine modulation through natural dietary compounds during early developmental stages are limited. To our knowledge, this is among the first studies to explore a potential dose–response relationship between dietary royal jelly supplementation and circulating estradiol levels in juvenile sterlets under controlled recirculating aquaculture conditions. Furthermore, the simultaneous evaluation of endocrine dynamics and somatic growth provides a multidimensional perspective on the biological effects of royal jelly, contributing novel insights into the development of natural strategies for endocrine regulation in sturgeon aquaculture.

The primary objective of the present study was to evaluate the effect of dietary royal jelly supplementation on endocrine-related responses in juvenile sterlets (*Acipenser ruthenus*), assessed through the determination of estradiol-related chromatographic responses by high-performance liquid chromatography (HPLC) in processed serum extracts. A secondary objective was to investigate potential correlations between these responses and a series of biometric parameters recorded at the group level. The biometric assessment included the determination of total length (L), standard length (Sl), maximum body height (H), body circumference (C), and body mass (BM).

The results obtained may contribute to the development of innovative functional feeds and to the implementation of physiological and productive control strategies in sturgeon aquaculture.

## 2. Results

### 2.1. Profile of 17β-Estradiol Chromatographic Responses Determined by HPLC

The chromatographic determination of 17β-estradiol in processed serum extracts revealed clear differences between the experimental variants. It should be emphasized that the reported values represent chromatographic concentrations in processed extracts (µg/mL extract) and are therefore intended for comparative interpretation, rather than direct representation of physiological serum estradiol levels (Table 1).

The control group exhibited low chromatographic responses, with a mean value of 5.57 ± 5.27 µg/mL extract. In contrast, all royal jelly-supplemented groups showed markedly higher values. The 1% supplementation variant (V1) reached a mean of 97.32 ± 73.74 µg/mL extract, corresponding to an approximately 17-fold increase relative to the control. The 3% variant (V2) showed a mean value of 110.13 ± 51.88 µg/mL extract, indicating sustained estrogen-associated chromatographic response with reduced variability compared to V1. The highest values were recorded in the 5% supplementation group (V3), with a mean of 185.31 ± 79.12 µg/mL extract, suggesting a clear dose-dependent effect.

The proportion of non-detectable (ND) samples also showed a clear decreasing trend with increasing supplementation. In the control group, 33.3% of samples were below the detection limit, whereas this proportion decreased to 16.7% in V1 and 6.7% in V2, while all samples in V3 showed detectable estradiol-related signals. This pattern further supports a stimulation of estrogen-related pathways.

In addition, the frequency of samples exhibiting high chromatographic responses (>200 µg/mL extract) increased in the higher supplementation variants. Such values were absent in the control group and limited in V1, but became more frequent in V2 and predominant in V3. This distribution shift toward higher response ranges suggests an intensification of estrogen-associated metabolic or endocrine activity.

For a more detailed characterization, values were grouped into three ranges: low (<50 µg/mL extract), moderate (50–150 µg/mL extract), and high (>200 µg/mL extract). The control group was predominantly characterized by non-detectable or low values, while V1 showed a wide distribution across all ranges. In V2, most values were concentrated in the moderate range, whereas V3 exhibited a substantial proportion of samples in the high range, together with the absence of ND values.

Although absolute quantification of physiological estradiol levels was not the objective of the present study, the observed dose-dependent increase in chromatographic response is consistent with the known role of estradiol in fish reproductive physiology. Previous studies have demonstrated that estrogenic responses can be effectively assessed through comparative analytical approaches, even when absolute quantification is limited by matrix effects or detection selectivity [14].

**Figure 1:**
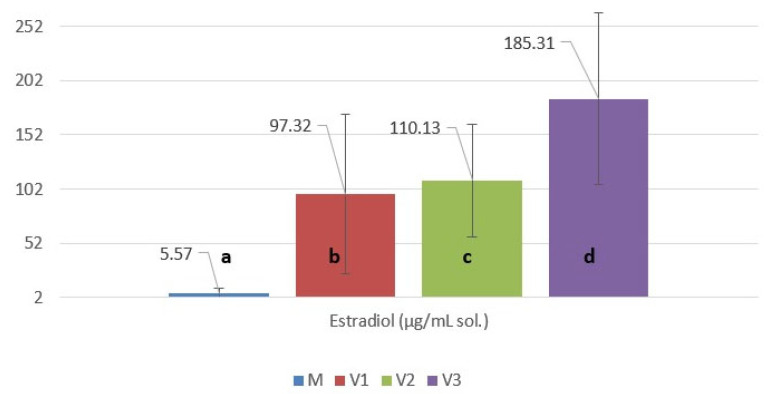
Mean values of 17β-estradiol chromatographic concentrations in processed serum extracts (µg/mL extract) Control variant (M)—commercial feed without royal jelly supplementation; Variant V1—feed supplemented with 1% royal jelly; Variant V2—feed supplemented with 3% royal jelly; Variant V3—feed supplemented with 5% royal jelly. Data with different letters are significantly different (*p* < 0.05).

Given the early developmental stage of the juveniles, individual variability was relatively high, particularly in the supplemented groups. Therefore, interpretation was conducted primarily at the group level, where consistent trends were observed. The overall results indicate a clear association between royal jelly supplementation and increased estradiol-related chromatographic response, suggesting a potential influence on endocrine pathways involved in sexual differentiation (Figure 1).

### 2.2. Results Regarding Biometric Assessment

**Figure 2:**
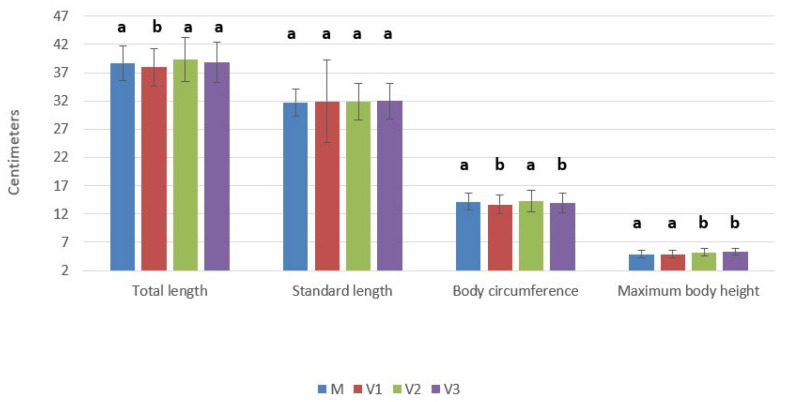
The mean values (*n* = 30) of total length, standard length, body circumference, and maximum body height. Control variant (M)—commercial feed without royal jelly supplementation; Variant V1—feed supplemented with 1% royal jelly; Variant V2—feed supplemented with 3% royal jelly; Variant V3—feed supplemented with 5% royal jelly. Data in the same group for each biometric parameter with different letters are significantly different (*p* < 0.05).

**Figure 3:**
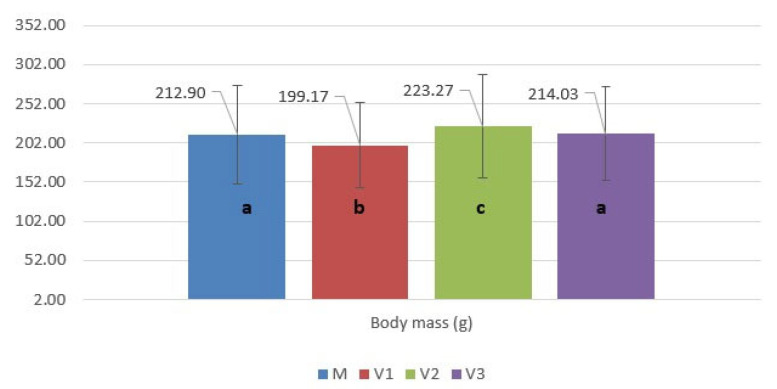
The mean body mass (g). Control variant (M)—commercial feed without royal jelly supplementation; Variant V1—feed supplemented with 1% royal jelly; Variant V2—feed supplemented with 3% royal jelly; Variant V3—feed supplemented with 5% royal jelly. Data with different letters are significantly different (*p* < 0.05).

The biometric parameters performed on 30 six-month-old juvenile sterlets (*Acipenser ruthenus*) are presented in the Supplementary Materials Tables S1 and S2. The biometric data presented in Figure 2 and Figure 3 indicate moderate differences in somatic growth parameters among the experimental variants. Mean total length (L) values were 38.63 ± 3.04 cm in the control group, 37.93 ± 3.28 cm in the 1% group, 39.30 ± 3.85 cm in the 3% group, and 38.85 ± 3.58 cm in the 5% group. The highest mean total length was recorded in the 3% supplementation variant, while the 1% group presented slightly lower values than the control.

Standard length (Sl) showed comparable mean values across treatments (31.70 ± 2.46 cm in M; 31.96 ± 7.30 cm in 1%; 31.84 ± 3.17 cm in 3%; and 31.95 ± 3.18 cm in 5%). Notably, the 1% group exhibited higher variability, as reflected by the elevated standard deviation (±7.30), indicating greater interindividual heterogeneity in this variant.

Maximum body height (H) demonstrated a clearer upward trend with increasing supplementation level, with mean values of 4.89 ± 0.62 cm (M), 4.89 ± 0.63 cm (1%), 5.25 ± 0.64 cm (3%), and 5.30 ± 0.56 cm (5%). The highest vertical development was therefore observed in the 5% royal jelly group.

Body circumference (C) presented mean values of 14.20 ± 1.52 cm in the control, 13.70 ± 1.73 cm in the 1% group, 14.33 ± 1.92 cm in the 3% group, and 13.91 ± 1.76 cm in the 5% group. The 3% variant showed the highest mean circumference, while the 1% group recorded slightly lower values compared to the control.

Regarding body mass (BM), mean values were 212.90 ± 62.53 g in the control group, 199.17 ± 53.96 g in the 1% variant, 223.27 ± 65.90 g in the 3% group, and 214.03 ± 60.16 g in the 5% group. The 3% royal jelly supplementation resulted in the highest mean body mass, whereas the 1% group showed lower average weight compared to the control.

Overall, growth performance did not follow a strictly linear dose–response pattern. The 3% supplementation level appeared to provide the most favorable results in terms of total length, body circumference, and body mass, while the 5% variant was associated with greater maximum body height. The relatively moderate variability observed in most parameters, except for standard length in the 1% group, supports the biological consistency of the experimental response. These findings suggest that royal jelly supplementation had limited influence on somatic development, although the effect appears to be moderate and parameter-specific rather than uniformly proportional to dose.

The statistical analysis of morphometric traits of the fish showed limited sensitivity to the applied treatments (Table 2). For total length, standard length, circumference, and body mass, the one-way ANOVA indicated no statistically significant differences among groups (all *p* > 0.05). Consistently, Tukey’s HSD post hoc test did not identify any significant pairwise contrasts (all adjusted *p*-values > 0.05). These findings suggest that, under the present experimental conditions, the treatments (1%, 3%, and 5%) did not induce measurable changes in overall growth performance relative to the control (M), and the variability observed within groups likely reflects normal biological variation among individuals rather than a systematic treatment effect.

In contrast, maximum height exhibited a significant overall group effect (one-way ANOVA, *p* = 0.0089), indicating that at least one group mean differed from the others when considering the global variance structure.

However, Tukey’s HSD comparisons (Table 3) remained non-significant after correction for multiple testing (all adjusted *p*-values > 0.05), with two contrasts showing borderline trends (M vs. 5%: *p* = 0.0524; 1% vs. 5%: *p* = 0.0508). This pattern typically occurs when the overall between-group variability is detectable, but individual pairwise differences are modest and/or within-group dispersion reduces the power once *p*-values are adjusted. Biologically, this may indicate a subtle shift in body depth (maximum height) at higher inclusion levels (5%), although the evidence is not strong enough to claim definitive pairwise differences at the conventional 0.05 threshold.

A critical data-quality aspect should be noted for standard length in the 1% group, (Supplementary Materials Table S1) where an extreme value (65.45) is present and markedly higher than the remaining observations. This outlier inflates the standard deviation and may mask potential differences. Therefore, verification of this value (e.g., transcription error vs. true measurement) is recommended. Nevertheless, the overall conclusion remains that the treatments primarily maintained comparable morphometric profiles, supporting the interpretation that fish size and condition were broadly similar across groups.

### 2.3. Correlation Between Biometric Parameters and Estradiol Chromatographic Concentrations in Processed Serum Extracts

**Figure 4:**
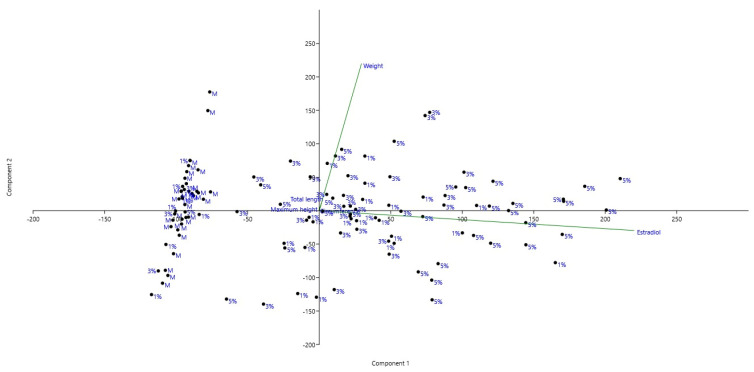
Correlation between biometric parameters and estradiol chromatographic concentrations in processed serum extracts.

Principal Component Analysis (PCA) was performed to explore the multivariate relationships between biometric parameters (total length, maximum height, and body mass) and estradiol chromatographic concentrations in processed serum extracts across experimental treatments (Figure 4).

The first principal component (PC1) explained 68.03% of the total variance, while the second principal component (PC2) accounted for 31.94%, indicating that these two components captured most of the variability within the dataset.

The loading vectors showed that estradiol strongly contributed to PC1, being oriented along the positive horizontal axis, whereas body mass was primarily associated with PC2, as indicated by its vertical orientation. In contrast, total length and maximum height were positioned near the origin of the biplot, suggesting a relatively minor contribution to the overall variability.

The spatial distribution of samples revealed a partial separation among treatments along PC1. Individuals from the 5% and 3% groups tended to be located toward the positive side of PC1, aligning with the estradiol vector, while the control (M) and 1% groups were predominantly clustered toward the negative region of this axis. This pattern suggests that higher inclusion levels may be associated with increased estradiol-related variability.

Furthermore, the orthogonal orientation between estradiol and weight vectors indicates that hormonal responses were not directly coupled with somatic growth. In other words, increases in estradiol chromatographic responsewere not proportionally associated with changes in body mass. Similarly, the weak contribution of total length and maximum height suggests that body shape parameters remained relatively stable across treatments.

Overall, the PCA highlights two independent biological processes: endocrine variability driven by estradiol and growth dynamics driven by body mass. These findings suggest that the experimental treatments may influence hormonal activity without inducing substantial morphological changes, while body mass variation follows a distinct pattern of regulation.

## 3. Discussion

The results obtained in the present study indicate that dietary supplementation with royal jelly modulates the endocrine profile of juvenile sterlets (*Acipenser ruthenus*), as reflected by the progressive increase in estradiol-related chromatographic responses determined by HPLC. It should be emphasized that the reported values represent concentrations in processed serum extracts (µg/mL extract) and are therefore interpreted as an estradiol-related chromatographic response rather than a definitive quantification of pure estradiol, given the limitations of UV-based detection without structural confirmation.

This progressive increase in chromatographic signal intensity with increasing royal jelly concentration supports the hypothesis of endocrine modulation. Similar trends have been reported in studies employing extraction-based HPLC-DAD methods for estrogenic compounds, where the analytical response reflects the processed extract and is influenced by extraction efficiency and matrix composition rather than representing direct biological concentrations [15,16].

A marked difference was observed between the control group (5.57 ± 5.27 µg/mL extract) and the supplemented variants (97.32 ± 73.74 µg/mL at 1%, 110.13 ± 51.88 µg/mL at 3%, and 185.31 ± 79.12 µg/mL at 5%), indicating a strong effect of royal jelly on estrogen-associated chromatographic signals. Although the increase was not strictly linear between intermediate doses, a clear upward trend was evident, with the highest response recorded in the 5% supplementation group.

The progressive reduction in non-detectable samples across experimental groups further supports the existence of a biologically relevant response. The decrease from 33.3% non-detectable values in the control group to complete detection in the 5% variant suggests an increasing activation of estrogen-related metabolic or endocrine pathways under the influence of royal jelly supplementation.

These findings are consistent with previous studies demonstrating that bioactive compounds, including peptides, fatty acids, and phenolic constituents present in natural supplements, can influence endocrine regulation and reproductive physiology in fish. Similar dose-dependent endocrine responses have been reported in nutritional studies involving functional feed additives, where modulation of steroidogenesis pathways was observed during early developmental stages [14,15].

At the same time, the interpretation of absolute estradiol values must consider the analytical limitations of the method. HPLC-DAD analysis in complex biological matrices is known to be susceptible to matrix effects and co-elution phenomena, particularly when detection is performed in the UV range (220–280 nm). Under such conditions, the measured signal may include contributions from structurally related or co-extracted compounds, potentially leading to an overestimation of absolute concentrations. This limitation has been widely reported in studies addressing estrogen determination in biological and environmental samples using HPLC-DAD [15].

Nevertheless, since all samples were processed and analyzed under identical conditions, the observed differences between experimental groups remain robust and biologically meaningful. Comparative approaches based on consistent analytical workflows are commonly employed in endocrine and nutritional studies, especially when investigating relative changes induced by dietary interventions rather than absolute hormone concentrations [14].

From a biological perspective, the increase in estradiol-associated responses, particularly in the higher supplementation variants, may indicate stimulation of pathways involved in ovarian differentiation. Estradiol plays a central role in fish gonadal development, and elevated levels during early life stages are associated with the activation of estrogen-dependent mechanisms. Therefore, the higher proportion of individuals exhibiting moderate and high chromatographic responses in the V2 and V3 groups may suggest a shift toward estrogen-dominated endocrine profiles, potentially influencing sex differentiation patterns.

However, given the early developmental stage of the studied juveniles, individual variability remains high, and endocrine responses should be interpreted primarily at the group level. Future studies employing more selective analytical techniques, such as LC–MS/MS, combined with internal standards and recovery correction, would allow accurate quantification of circulating estradiol levels and a more precise characterization of the observed endocrine effects [17]. Royal jelly contains proteins, fatty acids (notably 10-HDA), flavonoids, and phenolic compounds with documented estrogen-like activity. These components may influence steroidogenic pathways, particularly through modulation of aromatase (cyp19a1), the key enzyme responsible for converting androgens into estrogens [18,19]. Recent molecular investigations in sturgeon species have further highlighted the central role of genes such as foxl2 and cyp19a1 in mediating estradiol-dependent ovarian differentiation. For example, Hu et al. [20] demonstrated ovarian-specific expression patterns of foxl2 in Dabry’s sturgeon and its functional association with estradiol signaling, reinforcing the molecular basis of estrogen-driven gonadal differentiation.

The low estradiol-related chromatographic responses observed in the control group are consistent with the early juvenile stage of sturgeon development, during which gonadal differentiation processes are still incipient [21,22]. In contrast, the markedly increased chromatographic signals recorded in the supplemented groups suggest an intensification of estrogen-associated pathways. Although the values are expressed as concentrations in processed extracts (µg/mL extract) and do not represent absolute physiological serum levels, the observed differences between experimental variants indicate a potential stimulation of ovarian differentiation mechanisms under the influence of royal jelly supplementation. Experimental studies on Siberian sturgeon have shown that estradiol-17β exposure during early developmental windows can effectively influence gonadal differentiation pathways [23], supporting the concept that endocrine modulation during critical stages may alter the trajectory of sexual development.

Given the established role of estradiol in ovarian differentiation, the higher frequency of moderate and high estradiol values in the 3% and particularly in the 5% variants suggests a potential endocrine shift consistent with ovarian pathway activation. Although individual phenotypic sex cannot be definitively inferred solely from circulating hormone levels at this stage, the endocrine profile observed suggests an increased probability of ovarian pathway activation in the higher supplementation groups.

From an applied perspective, these findings are particularly relevant for sturgeon aquaculture. Females represent the economically valuable component of production systems due to their capacity to produce caviar. Advances in genetic sex identification and the development of WW superfemales [24] illustrate the growing interest in controlled sex management strategies. However, nutritional modulation offers a potentially less invasive and more sustainable complementary approach. In this context, royal jelly supplementation may represent a promising natural alternative to synthetic hormonal treatments traditionally used for sex control.

In addition to endocrine modulation, the present study evaluated biometric growth parameters. A limitation of the present study is that actual feed intake was not directly quantified. Feeding was standardized at 2% of biomass, a level selected based on preliminary observations conducted prior to the experiment, during which this ration resulted in rapid consumption with no visible uneaten feed. During the experimental period, no uneaten feed was visibly detected following feeding, suggesting a high level of feed utilization. However, differences in feed intake among experimental groups, including potential effects of diet palatability, cannot be excluded. Consequently, uniform or complete feed consumption cannot be confirmed. This limitation should be taken into account when interpreting both growth performance and endocrine responses, as possible variations in intake may have contributed to the observed outcomes.

Royal jelly supplementation showed limited and parameter-specific effects on somatic development. The 3% supplementation level showed in the highest mean total length, body circumference, and body mass, whereas the 5% group exhibited the greatest maximum body height. Growth performance did not follow a strictly linear dose–response pattern, suggesting a possible tendency toward improved nutrient utilization, although this effect was not statistically confirmed.

This partial dissociation between maximal estradiol levels (observed at 5%) and maximal weight gain (observed at 3%) indicates that endocrine modulation and somatic growth are not perfectly parallel processes during early juvenile development. Such divergence is biologically plausible, as energy allocation during early ontogenesis may prioritize regulatory and differentiation processes over pure biomass accumulation. Similar non-linear responses to natural feed additives have been reported in aquaculture species [18,19,25], where moderate inclusion levels improve metabolic efficiency, while excessive levels may shift physiological priorities.

The relatively high interindividual variability observed in estradiol concentrations, especially in the supplemented groups, reflects the physiological heterogeneity characteristic of early developmental stages [18]. Hormonal responsiveness may be influenced by genetic background, timing of gonadal differentiation, and metabolic adaptation to dietary components. Therefore, interpretation at the experimental group level remains both biologically and statistically appropriate.

Methodologically, HPLC proved suitable for identifying general endocrine trends among treatments. Nevertheless, as emphasized in classical endocrinological studies [24], integration with complementary techniques—such as gonadal histology and gene expression analysis (foxl2, cyp19a1, dmrt1, amh)—would provide a more comprehensive mechanistic understanding of the observed hormonal modulation.

From a practical perspective, the large-scale application of royal jelly in aquaculture may be limited by its availability and relatively high cost, as it is primarily used in high-value sectors such as pharmaceutical and nutraceutical industries. However, its potential economic relevance should also be considered in relation to its possible influence on sex differentiation. In sturgeon aquaculture, males have limited long-term economic value, whereas females are essential for caviar production. Therefore, even a moderate increase in the proportion of females could significantly enhance production efficiency and economic return. In this context, the use of royal jelly may be more feasible in targeted applications, such as early developmental stages or high-value production systems. Future studies should focus on optimizing inclusion levels, exploring bioactive fractions, and evaluating cost–benefit aspects under commercial conditions.

Although royal jelly represents a natural alternative to synthetic hormonal treatments, its endocrine-modulating activity warrants careful consideration from an environmental perspective. Estrogenic compounds, regardless of their origin, may act as endocrine disruptors in aquatic ecosystems if released into the environment. Therefore, the natural origin of royal jelly does not inherently eliminate potential ecological risks. However, natural products may differ from synthetic hormones in terms of persistence, biodegradability, and compound complexity, although these aspects require further investigation. Future studies should evaluate the environmental fate, residual estrogenic activity, and potential effects on non-target organisms under aquaculture conditions.

Overall, the present study demonstrates that royal jelly supplementation exerts a measurable endocrine effect in juvenile sterlet, with limited and parameter-specific effects on somatic growth performance. By integrating endocrine and biometric evaluation, this work contributes novel insights into the potential application of apicultural products as natural modulators of physiological and reproductive processes in sturgeon aquaculture.

## 4. Materials and Methods

The experimental study was designed and conducted in two distinct components, corresponding to the on-farm fish rearing phase and the subsequent laboratory analysis phase, carried out sequentially.

### 4.1. Experimental Stages in the Fish Farm

The on-farm component of the experiment was conducted at the Aquaculture Research Laboratory of the University of Life Sciences “King Mihai I of Romania” in Timișoara. The system consisted of four rectangular fiberglass tanks, each with a useful volume of approximately 1 m^3^, as well as four additional tanks with a capacity of 8 m^3^. The installation was equipped with mechanical and biological filtration units, aeration systems, and environmental parameter control devices, ensuring optimal conditions for sturgeon rearing [26].

The biological material consisted of six-month-old juvenile sterlets (*Acipenser ruthenus*). Sexual differentiation begins at approximately this age [27], and the administration of royal jelly could potentially favor an increased proportion of females. The specimens originated from broodstock maintained exclusively in recirculating aquaculture systems and were obtained through artificial reproduction during the 2024 breeding season. Prior to the experiment, the juveniles were fed commercial granulated feeds formulated for sturgeons, supplied by Alltech Coppens (Helmond, The Netherlands).

During the experimental period, the commercial feed SUPREME–10 (Alltech Coppens (Helmond, The Netherlands)), containing 49% crude protein and 10% lipids, was used as the basal diet. This formulation is designed to support rapid and balanced sturgeon development. To evaluate the effect of royal jelly on biological parameters, the basal feed was supplemented with this apicultural product, recognized for its high nutritional value and reported beneficial effects on growth and animal physiology, according to the scientific literature [7].

**Figure 5:**
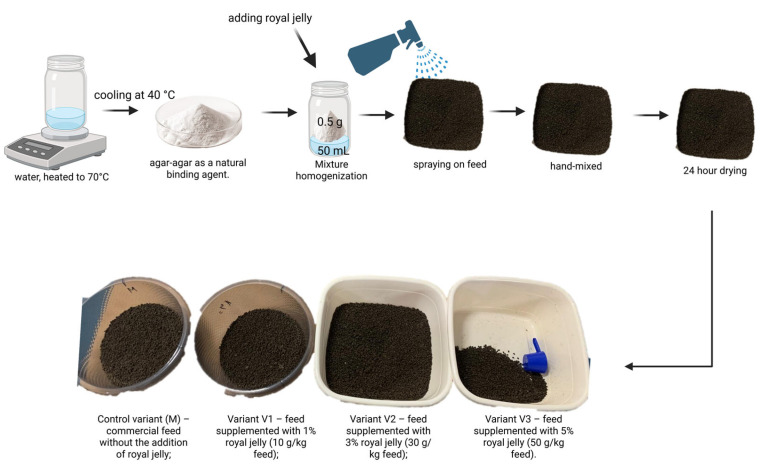
Illustrates the technological process of feed supplementation with royal jelly (Figure created with BioRender.com, accessed on 23 January 2026).

To ensure uniform adhesion of royal jelly to the 3 mm feed pellets, agar-agar was used as a natural binding agent. Agar was dissolved at a ratio of 0.5 g per 50 mL of water and heated to approximately 70 °C to allow complete dissolution of the powder. After cooling the solution to approximately 40 °C, royal jelly was added in amounts corresponding to each experimental concentration and homogenized thoroughly. The resulting mixture was uniformly sprayed onto the feed pellets distributed in a thin layer on a flat surface. The pellets were then manually mixed and left to dry at room temperature for 24 h to stabilize the freshly prepared feed (Figure 5).

Royal jelly used in the experiment was obtained from a commercial source and stored under refrigerated conditions until use. The royal jelly used in this study originated from the same commercial batch previously characterized in detail in our earlier studies [5,28], including physicochemical composition and bioactive compound profiling by LC-MS.

**Figure 6:**
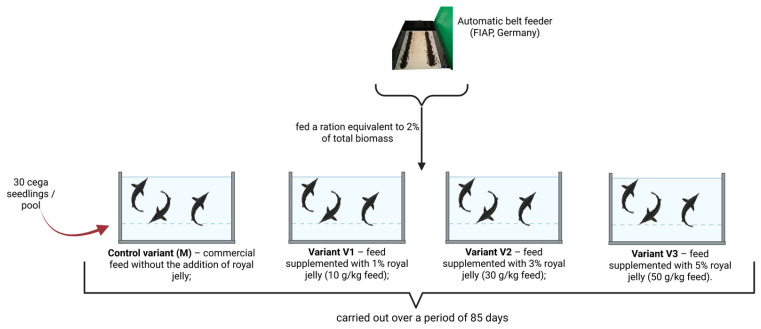
Illustrates the experimental groups (Figure created with BioRender.com, accessed on 23 January 2026).

The experimental design included four nutritional variants, each consisting of 30 juvenile sterlet maintained in a separate tank. SUPREME–10 feed was used as the basal diet in all variants, the differentiation being achieved through the level of royal jelly supplementation, as follows (Figure 6):

➢ Control variant (M)—commercial feed without royal jelly supplementation;
➢ Variant V1—feed supplemented with 1% royal jelly (10 g/kg feed);
➢ Variant V2—feed supplemented with 3% royal jelly (30 g/kg feed);
➢ Variant V3—feed supplemented with 5% royal jelly (50 g/kg feed).

The experiment was conducted over a period of 85 days. Fish were fed daily with a ration equivalent to 2% of the total biomass in each tank, and the weight of each group was recorded biweekly. Feed was administered continuously over a 24 h period using an automatic belt feeder FIAP GmbH (Ursensollen, Germany), thereby ensuring a constant and uniform distribution of feed.

Throughout the experimental period, the main physico-chemical water parameters were periodically monitored and maintained within ranges suitable for juvenile sterlet rearing. The recorded values were as follows: water temperature 17.0–20.1 °C, pH 7.20–8.20, electrical conductivity 367.6–377.6 μS/cm, turbidity 1.51–1.61 NTU, chlorides 13.6–14.6 mg/L, total calcium and magnesium ions 9.06–10.06 mg/L, total iron 47.55–57.55 μg/L, manganese 35.8–45.8 μg/L, ammonium 0.29–0.39 mg/L, nitrates 29.44–30.44 mg/L, nitrites 0.42–0.52 mg/L, oxidability 2.17–3.17 mg O_2_/L, and sulfates 69.04–71.05 mg/L.

To maintain water quality in the recirculating system, the recirculation flow rate was adjusted so that the water in each tank was renewed approximately twice per hour. In addition, approximately 15% of the total system water volume was replaced daily with fresh water.

At the end of the experimental period, biological samples were collected for hormonal analysis, and the specimens included in the study were subjected to biometric evaluation according to the methods described by Bura [26]. The biometric assessment included the determination of total length (L), standard length (Sl), maximum body height (H), body circumference (C), and body mass (BM).

### 4.2. Determination of Steroid Levels

#### 4.2.1. Collection and Preservation of Biological Samples

For hormonal analysis, approximately 1.5 mL of blood was collected from each selected specimen by venipuncture at the level of the caudal vein, using sterile syringes and needles, following standard procedures described for teleost and sturgeon species [29]. To minimize stress-induced endocrine interference, fish handling time was reduced, and sampling was performed under controlled conditions, as recommended in fish endocrinology protocols [30].

The blood samples were allowed to clot at room temperature for approximately 30 min and were subsequently centrifuged at 3000 rpm for 10 min to separate the serum, according to established endocrine sampling methodologies [31].

The obtained serum was transferred into sterile microtubes and stored at −50 °C until chromatographic analysis to prevent degradation of steroid hormones and preserve sample stability [31,32]. Proper low-temperature storage is essential to maintain steroid integrity prior to analytical quantification [32].

#### 4.2.2. Sample Preparation and Steroid Extraction

For steroid extraction, a volume of 0.7 mL of serum was transferred into 15 mL centrifuge tubes. An equal volume of acetonitrile (0.7 mL) was added to each sample as an organic solvent for steroid compound extraction, following commonly used liquid–liquid extraction protocols for fish steroid hormones [31,33]. The mixture was homogenized using a vortex mixer (Vortex-Genie 2, model G-560E (Scientific Industries, Bohemia, NY, USA)) for 1–2 min to ensure efficient extraction of lipophilic steroid compounds.

Subsequently, the samples were centrifuged using a Hettich EBA 21 laboratory centrifuge (Hettich Zentrifugen (Tuttlingen, Germany) at 3000 rpm for 10 min to achieve phase separation. After centrifugation, the organic extract (liquid fraction) was collected and subjected to a concentration step. The resulting residue was re-dissolved in a mixture of acetonitrile and distilled water (0.7 mL:0.7 mL), vortexed for 2 min, and filtered prior to chromatographic analysis.

Prior to injection into the HPLC all preparations were homogenized using an ultrasonic bath (Elma, model E 100 H (Elma Schmidbauer GmbH, Singen, Germany), ensuring complete dissolution of analytes and improved chromatographic reproducibility [33].

#### 4.2.3. HPLC Analysis

The analytical method was designed for comparative evaluation of estradiol-related chromatographic responses between experimental groups. Estradiol identification was performed using an external standard of analytical-grade 17β-estradiol, based on matching retention time and UV spectral characteristics recorded by the diode array detector. Chromatographic analysis was performed using a HPLC system equipped with a diode array detector Shimadzu Corporation (Kyoto, Japan) and reverse-phase C18 column (particle size 3 µm). The mobile phase consisted of methanol, acetonitrile, and water (50:30:20, *v*/*v*/*v*), optimized for estradiol separation. The mobile phase flow rate was set at 1.0 mL/min. Detection of steroid compounds was carried out using a UV detector Shimadzu Corporation (Kyoto, Japan) within a wavelength range of 220–280 nm, consistent with previously validated HPLC methods [33]. The injection volume was 40 µL, and the retention time specific to estradiol was approximately 1.83 min. The total duration of each chromatographic run was 5 min per sample.

Estradiol identification was performed using an external calibration standard of pure estradiol, and the analyte was assigned based on matching retention time and chromatographic signal characteristics.

The reported estradiol values are expressed as µg/mL of processed extract obtained after sample preparation. These values reflect chromatographic concentrations in the analytical phase and are used for comparative purposes between experimental groups, rather than representing absolute physiological serum concentrations.

The analytical performance of the HPLC method for estradiol determination was evaluated in terms of linearity, sensitivity, and repeatability. The calibration curve was constructed using standard solutions of estradiol, yielding the regression equation y = 1.8592 × 10^−4^x − 4.46634, with a correlation coefficient (R^2^) of 0.9874.

The sensitivity of the method was assessed by calculating the limit of detection (LOD) and limit of quantification (LOQ), according to the International Conference on Harmonisation (ICH) guidelines. Based on the standard deviation of the response (σ = 2.435031 × 10^−5^) and the slope of the calibration curve (S = 1.8592 × 10^−4^), the LOD and LOQ were estimated as 0.43 µg/m L and 1.31 µg/m L, respectively. These values indicate that the method is suitable for the detection and quantification of estradiol at low concentration levels in biological matrices.

Elevated 17β-estradiol concentrations were considered indicative of activation of ovarian differentiation pathways, in accordance with established models of endocrine control in fish [18,33].

Given the developmental stage of the juveniles, the interpretation of hormonal results was conducted primarily at the experimental group level through comparison of mean hormonal values between the control variant and the royal jelly-supplemented variants. This approach is consistent with recommendations for endocrine studies in early developmental stages, where individual hormone levels may exhibit high variability and low absolute concentrations [31,33].

### 4.3. Biometric Assessment

Biometric evaluation was conducted to determine somatic growth parameters in juvenile sterlets (*Acipenser ruthenus*) subjected to different levels of royal jelly supplementation. At the end of the experimental period, 30 individuals from each experimental variant were analyzed.

Fish handling and measurements were performed under conditions designed to minimize physiological stress, considering that stress may influence metabolic and endocrine parameters [31,32]. Biometric determinations were carried out on a moist measuring surface to prevent skin damage and dehydration.

Total length (L, cm) was measured from the tip of the snout to the end of the caudal fin, while standard length (Sl, cm) was recorded from the snout to the base of the caudal peduncle, in accordance with standard methodologies used in fish growth studies [34]. Maximum body height (H, cm) was determined at the greatest dorsal–ventral distance using a digital caliper with a precision of 0.01 cm. Body circumference (C, cm) was measured at the widest region of the trunk using a flexible measuring tape.

Body mass (BM, g) was determined individually using a calibrated electronic balance with an accuracy of 0.01 g. All measurements were completed within a short time interval to reduce handling effects on physiological parameters [33].

The selected biometric parameters allowed for the assessment of longitudinal growth (L, Sl), vertical development (H), transverse development (C), and overall somatic performance (W). Such measurements are commonly applied in aquaculture nutrition and growth studies to evaluate productive performance and morphological development [34,35]. The collected data were used to calculate mean values and standard deviations for each experimental group (*n* = 30).

The applied procedures ensured accuracy and reproducibility of biometric determinations and are consistent with protocols employed in contemporary fish physiology and nutrition research.

### 4.4. Statistical Analysis

Data are expressed as mean ± standard deviation (SD). Differences among experimental groups were analyzed by one-way analysis of variance (ANOVA), followed by Tukey’s post hoc test for multiple comparisons. Statistical analyses were performed using JASP software (version 0.95.4). A *p*-value < 0.05 was considered statistically significant. Thirty individuals were analyzed per treatment group.

## 5. Conclusions

The results obtained in the present study demonstrate that dietary supplementation with royal jelly significantly influences the endocrine profile of a juvenile sterlet (*Acipenser ruthenus*), as reflected by increased estradiol-related chromatographic responses determined by HPLC. Compared with the control group, all supplemented experimental variants exhibited higher mean values, suggesting hormonally mediated modulation induced by this natural apicultural product.

The observed dose-dependent increase in chromatographic responses supports the potential of royal jelly as a functional feed additive capable of influencing endocrine-related processes during early developmental stages. However, further studies using more selective analytical techniques are required to confirm these effects at the level of physiological hormone concentrations.

In conclusion, the data suggest that royal jelly represents a promising natural alternative to synthetic hormonal substances, with potential application in nutritional strategies aimed at influencing sexual differentiation in sturgeon aquaculture. The HPLC-based approach proved useful for evaluating endocrine-related trends at the group level; however, for precise individual assessment and validation of sex differentiation, complementary methods such as gonadal histology or gene expression analysis of key markers involved in sexual differentiation are recommended.

The findings of the present study contribute to expanding current knowledge on the use of natural feed additives in aquaculture and align with recent trends promoting functional nutrition as a tool for physiological and reproductive regulation in fish. Contemporary research highlights the role of bioactive natural compounds in modulating endocrine, immune, and metabolic status, with positive implications for productive performance and organism resilience [35,36]. In this context, the incorporation of apicultural products, including royal jelly, supports modern strategies aimed at reducing dependence on synthetic hormones and chemical additives, thereby fostering more sustainable and consumer-acceptable production systems. The integration of such natural solutions into feed formulations opens new perspectives for the development of innovative functional feeds targeting sex differentiation control and reproductive optimization, in accordance with the principles of sustainable aquaculture and animal welfare.

## Figures and Tables

**Table 1:** Individual 17β-estradiol chromatographic concentrations in processed serum extracts (µg/mL extract) in juvenile sterlets (*Acipenser ruthenus*) across experimental groups (*n* = 30 per group). M—control group; V1—1% royal jelly; V2—3% royal jelly; V3—5% royal jelly. * ND—non-detectable (below the detection limit of the method).

Sample	M	V1	V2	V3
**1**	12.03 ± 0.01	273.52 ± 0.01	141.68 ± 0.01	106.16 ± 0.03
**2**	7.34 ± 0.02	17.08 ± 0.02	101.45 ± 0.02	232.59 ± 0.01
**3**	10.88 ± 0.01	ND *	100.00 ± 0.01	180.00 ± 0.03
**4**	ND	125.66 ± 0.01	113.39 ± 0.01	278.62 ± 0.01
**5**	6.79 ± 0.02	ND	120.10 ± 0.03	137.47 ± 0.02
**6**	ND	101.10 ± 0.02	102.02 ± 0.01	102.89 ± 0.01
**7**	8.00 ± 0.01	ND	124.24 ± 0.03	71.28 ± 0.01
**8**	5.30 ± 0.01	ND	121.98 ± 0.03	52.81 ± 0.01
**9**	5.31 ± 0.02	1.81 ± 0.01	129.06 ± 0.01	216.54 ± 0.03
**10**	9.45 ± 0.01	206.84 ± 0.02	183.96 ± 0.01	53.82 ± 0.01
**11**	ND	93.48 ± 0.01	47.44 ± 0.02	211.21 ± 0.03
**12**	ND	157.58 ± 0.01	2.01 ± 0.01	83.07 ± 0.01
**13**	ND	127.13 ± 0.01	192.10 ± 0.03	189.38 ± 0.01
**14**	ND	168.80 ± 0.02	156.40 ± 0.01	266.32 ± 0.03
**15**	ND	203.07 ± 0.01	ND	196.74 ± 0.03
**16**	ND	1.74 ± 0.01	184.74 ± 0.01	214.07 ± 0.01
**17**	ND	139.96 ± 0.03	156.01 ± 0.02	121.47 ± 0.01
**18**	7.42 ± 0.01	95.67 ± 0.01	125.45 ± 0.01	266.02 ± 0.03
**19**	6.12 ± 0.01	154.80 ± 0.01	ND	6.90 ± 0.01
**20**	2.41 ± 0.02	120.22 ± 0.01	115.66 ± 0.01	243.01 ± 0.01
**21**	8.48 ± 0.01	ND	153.95 ± 0.01	298.01 ± 0.01
**22**	ND	123.15 ± 0.01	112.34 ± 0.01	172.74 ± 0.03
**23**	5.16 ± 0.01	146.56 ± 0.01	86.73 ± 0.03	249.3 ± 0.01
**24**	4.76 ± 0.03	96.95 ± 0.01	153.66 ± 0.01	272.79 ± 0.01
**25**	7.51 ± 0.01	81.75 ± 0.02	118.63 ± 0.03	301.77 ± 0.02
**26**	5.32 ± 0.01	115.00 ± 0.01	69.69 ± 0.01	230.66 ± 0.01
**27**	16.98 ± 0.02	97.46 ± 0.01	156.76 ± 0.01	191.12 ± 0.03
**28**	10.23 ± 0.01	127.4 ± 0.01	79.42 ± 0.03	224.78 ± 0.01
**29**	7.10 ± 0.01	2.26 ± 0.03	92.46 ± 0.01	192.43 ± 0.01
**30**	20.42 ± 0.02	142.94 ± 0.01	42.62 ± 0.01	195.47 ± 0.01
**Mean**	5.57 ± 5.27	97.32 ± 73.74	110.13 ± 51.88	185.31 ± 79.12

Values are expressed as mean ± SD.

**Table 2:** The statistical analysis of morphometric traits.

Parameter	M (*n* = 30)	1% (*n* = 30)	3% (*n* = 30)	5% (*n* = 30)	*p* (ANOVA)
Total length	38.63 ± 3.04	37.93 ± 3.28	39.29 ± 3.85	38.85 ± 3.58	0.488
Standard length	31.70 ± 2.46	31.96 ± 7.30	31.84 ± 3.17	31.95 ± 3.18	0.996
Maximum height	4.89 ± 0.62	4.89 ± 0.63	5.25 ± 0.64	5.30 ± 0.56	0.0089
Circumference	14.25 ± 1.52	13.68 ± 1.73	14.45 ± 1.92	14.05 ± 1.76	0.359
Body mass	212.92 ± 62.53	199.31 ± 53.96	223.67 ± 65.90	214.32 ± 60.16	0.487

**Table 3:** Tukey’s HSD post hoc test results for pairwise comparisons of morphometric parameters among experimental fish groups.

Parameters	M vs. 1%	M vs. 3%	M vs. 5%	1% vs. 3%	1% vs. 5%	3% vs. 5%
Total length	0.8629	0.8760	0.9947	0.4216	0.7333	0.9579
Standard length	0.9960	0.9993	0.9964	0.9996	1.0000	0.9997
Maximum height	1.0000	0.1005	0.0524	0.0976	0.0508	0.9929
Circumference	0.5827	0.9682	0.9711	0.3138	0.8401	0.8057
Body mass	0.8216	0.9028	0.9997	0.4102	0.7742	0.9333

## Data Availability

The report of the analyses performed for the samples in the paper can be found on the “Interdisciplinary Research Platform” of the University of Life Sciences “King Mihai I” from Timișoara.

## Supplementary Materials

The following supporting information can be downloaded at: https://www.mdpi.com/article/10.3390/molecules31071210/s1, Table S1: Suplementary Material 1. The biometrics parameters: total, standard and maximum lengths (cm) for 30 six-month-old juvenile sterlet (*Acipenser ruthenus*), Table S2: Suplementary Material 2. The biometrics parameters: circumference (cm) and body mass (g) for 30 six-month-old juvenile sterlet (*Acipenser ruthenus*).
