# Supplementary material for: Royal Jelly as a Natural Endocrine Modulator of Serum Estradiol Levels in Juvenile Sterlets (Acipenser ruthenus)
Source: Molecules. 2026 Apr 6;31(7):1210. doi: 10.3390/molecules31071210 (PMC13074310; doi:10.3390/molecules31071210)
Supplement: Supplementary file 1 [file molecules-31-01210-s001.zip › Suplementary Material 1_SM1.pdf]

Table S1. The biometrics parameters: total, standard and maximum lengths (cm) for *30* six-month-old juvenile sterlet (Acipenser ruthenu*s)*

Sample	Total length				Standard length				Maximum height			
	M	1%	3%	5%	M	1%	3%	5%	M	1%	3%	5%
**1**	39.40±1.01	34.67±0.57	41.24±0.84	40.63±0.57	32.83±0.57	29.17±0.59	35.33±0.75	32.23±0.59	4.76±0.10	4.66±0.10	5.62±0.04	5.39±0.23
**2**	40.90±1.33	36.93±0.59	41.53±1.11	40.33±0.59	34.27±0.69	29.83±0.59	33.17±1.15	33.23±0.57	5.37±0.05	5.38±0.03	5.12±0.11	5.40±0.16
**3**	34.10±0.62	38.47±0.57	44.23±0.57	34.33±0.41	32.16±0.79	32.34±0.28	35.19±0.53	24.83±0.57	5.27±0.05	4.80±0.16	5.88±0.05	4.53±0.22
**4**	39.17±1.26	42.83±0.04	41.07±1.02	40.73±0.22	31.46±0.39	65.45±0.10	32.17±1.15	34.23±0.59	4.87±0.11	5.45±0.08	5.26±0.11	5.80±0.01
**5**	37.67±1.01	29.38±0.15	39.83±0.57	42.86±0.06	31.73±0.25	24.17±0.57	32.73±0.59	36.23±0.59	4.42±0.12	3.40±0.01	4.97±0.12	6.37±0.12
**6**	38.40±1.01	32.41±0.16	39.53±0.40	44.19±0.67	30.57±0.89	26.17±0.59	32.17±1.16	35.25±0.62	5.32±0.22	3.77±0.11	5.10±0.01	5.97±0.12
**7**	39.00±1.05	41.31±0.36	38.13±0.40	40.24±0.54	31.40±0.60	43.80±0.01	31.53±0.40	32.73±0.56	5.25±0.07	5.63±0.07	5.25±0.08	5.52±0.22
**8**	39.93±1.35	40.53±0.57	39.23±0.57	28.83±0.21	33.30±0.36	32.50±0.01	31.27±1.15	23.82±0.55	5.20±0.01	5.41±0.01	5.33±0.06	3.70±0.01
**9**	35.47±0.23	40.33±0.59	35.27±0.45	39.77±0.12	29.00±0.68	32.83±0.57	29.83±0.59	32.20±0.69	4.77±0.06	5.42±0.21	5.39±0.04	5.87±0.12
**10**	38.53±0.40	39.20±0.01	41.00±0.16	40.65±0.23	33.60±1.01	32.04±0.95	32.67±0.28	32.13±0.57	5.32±0.12	5.27±0.11	5.42±0.06	5.60±0.01
**11**	40.80±0.01	37.93±0.57	41.56±0.44	37.60±0.01	32.10±0.52	31.53±0.59	32.66±0.28	31.13±0.59	5.40±0.01	4.90±0.18	5.40±0.01	5.40±0.16
**12**	40.87±0.59	36.87±0.11	39.59±0.34	35.23±0.57	31.84±1.16	23.50±0.01	32.66±0.27	29.80±0.18	5.73±0.24	4.51±0.16	5.46±0.10	5.56±0.26
**13**	40.20±0.01	38.27±0.57	43.80±1.72	42.70±0.34	32.97±0.57	31.13±0.57	34.74±0.25	34.60±0.19	4.80±0.01	5.32±0.40	5.54±0.23	5.50±0.16
**14**	36.33±0.11	40.73±0.57	46.97±1.15	41.72±0.22	29.53±0.33	33.13±0.59	38.47±0.58	34.67±0.28	4.55±0.10	5.37±0.28	6.49±0.31	5.60±0.01
**15**	37.70±0.7	39.03±0.59	38.13±1.45	41.31±0.55	30.97±0.51	31.47±0.45	29.81±0.27	34.63±0.22	4.70±0.01	4.67±0.12	4.93±0.07	5.65±0.10
**16**	43.54±0.23	40.23±0.57	42.07±1.16	41.83±0.41	34.17±0.57	32.17±0.57	33.93±0.57	35.13±0.57	5.14±0.23	4.69±0.14	5.85±0.04	5.86±0.10
**17**	42.80±0.01	31.70±0.11	39.70±0.18	36.62±0.22	34.67±0.16	28.57±0.59	32.97±1.15	31.70±0.51	5.22±0.03	5.53±0.24	5.28±0.10	5.25±0.25

**18**	36.67±0.62	41.74±0.46	31.63±1.11	39.85±0.10	30.63±0.14	33.63±0.59	25.97±0.58	32.83±0.57	4.42±0.04	5.80±0.16	3.90±0.01	5.45±0.07
**19**	37.60±0.60	36.87±0.59	32.18±0.32	38.90±0.01	30.62±0.81	29.50±0.01	26.50±0.01	31.84±0.60	4.67±0.12	4.63±0.20	4.36±0.10	4.93±0.06
**20**	45.10±0.19	43.07±0.57	39.23±0.57	39.79±0.17	36.27±0.68	34.83±0.57	31.63±1.00	31.87±0.11	5.73±0.07	5.03±0.12	5.43±0.07	5.48±0.15
**21**	39.37±1.16	36.27±0.59	44.22±0.39	40.31±0.36	31.93±0.51	29.83±0.57	37.80±0.01	33.96±0.78	4.65±0.09	3.71±0.14	6.47±0.13	5.40±0.01
**22**	44.47±1.52	38.67±0.28	41.53±0.39	41.40±0.35	36.10±0.54	31.73±0.57	33.03±0.22	34.65±0.59	6.42±0.02	5.24±0.08	5.78±0.13	5.37±0.14
**23**	37.00±0.01	38.34±1.15	40.49±0.33	38.29±0.50	33.00±0.51	31.93±0.59	33.23±0.57	31.66±0.28	4.80±0.16	5.32±0.19	5.77±0.12	4.80±0.01
**24**	34.46±0.08	36.50±0.01	35.61±0.36	35.05±0.25	27.27±0.69	29.10±0.01	28.77±0.23	31.62±0.22	3.85±0.09	4.44±0.06	5.21±0.21	4.85±0.09
**25**	33.60±1.01	35.89±0.20	37.71±0.27	42.63±0.21	26.30±0.01	28.93±0.57	29.63±0.22	34.80±0.01	3.63±0.11	4.45±0.08	4.86±0.11	5.77±0.13
**26**	33.40±1.00	32.33±0.57	42.22±0.57	40.20±0.53	26.16±0.93	25.17±0.59	33.70±0.17	34.10±0.68	3.62±0.03	3.66±0.10	5.74±0.06	5.36±0.12
**27**	39.47±0.36	40.21±0.38	36.21±0.37	32.67±0.16	32.33±0.75	31.57±0.47	29.93±0.51	26.93±0.74	5.27±0.12	5.22±0.20	4.78±0.14	4.47±0.14
**28**	37.97±0.57	39.46±0.45	29.31±0.37	39.01±0.11	31.64±0.14	31.10±0.36	23.76±0.23	31.59±0.15	4.33±0.11	4.67±0.22	3.47±0.11	4.93±0.05
**29**	35.19±0.53	39.26±0.09	37.25±0.54	35.35±0.52	29.34±0.28	31.17±0.59	29.80±0.26	28.37±0.22	4.25±0.07	4.77±0.07	4.70±0.18	4.42±0.03
**30**	39.66±0.11	38.46±0.27	38.38±0.47	32.35±0.59	32.75±0.24	30.37±0.76	30.12±0.55	25.63±0.24	4.93±0.05	5.48±0.13	4.85±0.07	4.70±0.16
**Mean**	38.63	37.93	39.30	38.85	31.70	31.96	31.84	31.95	4.89	4.89	5.25	5.30
**SD**	3.04	3.28	3.85	3.58	2.46	7.30	3.17	3.18	0.62	0.63	0.64	0.56

Values are expressed as mean ± standard deviation (n = 30).
