# Supplementary material for: Royal Jelly as a Natural Endocrine Modulator of Serum Estradiol Levels in Juvenile Sterlets (Acipenser ruthenus)
Source: Molecules. 2026 Apr 6;31(7):1210. doi: 10.3390/molecules31071210 (PMC13074310; doi:10.3390/molecules31071210)
Supplement: Supplementary file 1 [file molecules-31-01210-s001.zip › Suplementary Material 2_SM2.pdf]

Table S2. The biometrics parameters: circumference (cm) and body mass (g) for *30* six-month-old juvenile sterlet (Acipenser ruthenus*)*

Sample	Circumference				Body mass			
	M	1%	3%	5%	M	1%	3%	5%
1	13.89±0.03	13.35±0.26	16.16±0.66	14.50±0.16	227.77±0.41	157.67±0.59	269.33±0.57	232.33±2.30
2	16.17±0.12	14.34±0.11	14.63±0.21	14.40±0.34	261.80±0.36	195.82±0.33	237.27±0.47	241.33±0.57
3	14.47±0.11	14.38±0.22	15.93±0.05	11.20±0.01	230.20±0.36	198.78±0.40	295.00±0.01	131.27±0.47
4	14.32±0.20	15.26±0.09	16.87±0.07	17.23±0.57	216.85±0.26	257.33±0.57	237.32±0.55	273.30±0.53
5	13.63±0.11	9.62±0.22	13.95±0.05	17.13±0.5	180.03±0.06	73.00±0.01	222.27±0.56	322.29±0.51
6	15.24±0.06	9.90±0.01	13.69±0.15	16.37±0.55	228.33±0.57	88.00±0.01	212.00±0.01	305.33±0.57
7	14.74±0.06	15.75±0.27	14.86±0.11	14.33±0.22	226.00±0.01	274.67±0.58	217.90±0.85	218.32±0.57
8	15.25±0.26	14.87±0.57	14.39±0.32	9.67±0.28	229.11±0.12	235.77±0.40	207.24±0.42	73.33±0.57
9	12.73±0.22	15.37±0.41	15.21±0.19	14.46±0.27	171.11±0.19	231.26±0.65	188.65±1.11	235.23±0.41
10	15.21±0.16	14.93±0.05	15.72±0.22	15.51±0.19	223.00±0.02	234.96±0.08	246.65±1.11	245.29±0.52
11	16.23±0.06	13.80±0.16	15.94±0.06	14.70±0.18	257.96±0.83	201.79±0.37	256.31±0.54	190.24±0.42
12	15.61±0.07	13.33±0.05	13.84±0.08	14.43±0.24	267.13±0.44	171.22±0.37	240.25±0.44	154.19±0.34
13	14.93±0.06	15.50±0.36	16.34±0.24	15.25±0.07	228.67±0.57	233.34±0.57	282.88±0.89	260.25±0.44
14	13.41±0.34	15.20±0.01	17.63±0.23	14.59±0.17	175.17±0.30	242.34±1.16	368.25±0.34	252.23±0.41
15	13.60±0.01	12.73±0.11	13.54±0.05	16.59±0.16	184.93±0.12	193.00±0.01	194.31±0.49	260.31±0.54
16	15.59±0.03	13.52±0.15	14.69±0.16	14.29±0.51	274.77±0.41	220.33±0.59	232.33±0.57	272.29±0.51
17	14.73±0.11	15.53±0.11	14.76±0.09	13.58±0.15	247.97±0.06	207.67±1.16	219.29±0.50	210.27±0.47
18	12.10±0.01	15.87±0.11	9.73±0.22	15.07±0.46	163.25±0.44	283.33±1.16	97.33±0.57	249.23±0.41

**19**	14.26±0.09	13.03±0.06	10.80±0.18	13.64±0.25	187.29±0.52	181.50±0.87	108.58±0.52	198.21±0.37
**20**	16.87±0.05	14.40±0.01	13.85±0.10	12.66±0.29	350.30±0.53	297.66±1.16	221.34±0.57	214.00±0.01
**21**	13.57±0.11	11.50±0.33	17.23±0.57	13.45±0.26	190.07±0.12	148.33±0.59	363.28±0.48	240.31±0.54
**22**	17.56±0.13	14.57±0.30	14.86±0.63	13.80±0.01	378.17±0.48	203.55±0.78	266.96±0.92	213.30±0.53
**23**	14.50±0.33	13.58±0.40	14.68±0.13	13.49±0.34	209.23±0.41	227.15±0.26	261.56±0.50	181.26±0.45
**24**	11.53±0.24	12.63±0.45	13.47±0.29	12.63±0.23	110.00±0.01	156.75±0.45	174.00±0.01	200.22±0.40
**25**	11.93±0.05	13.67±0.24	13.57±0.12	15.57±0.12	102.67±0.59	161.00±6.94	181.56±0.50	288.00±0.01
**26**	11.40±0.36	9.40±0.16	16.39±0.31	13.79±0.16	90.67±0.59	84.34±0.57	283.28±0.50	230.30±0.53
**27**	14.55±0.38	13.36±0.27	13.67±0.28	13.47±0.30	218.67±0.59	195.34±0.57	154.66±0.57	120.32±0.56
**28**	12.87±0.11	12.66±0.26	9.47±0.28	12.70±0.18	190.96±0.95	201.18±0.33	69.32±0.57	180.29±0.52
**29**	12.76±0.08	14.47±0.30	13.80±0.01	12.53±0.24	135.33±0.57	218.21±0.36	197.31±0.53	145.24±0.42
**30**	13.82±0.03	13.82±0.15	13.95±0.05	10.49±0.33	230.25±0.44	203.90±0.16	203.58±0.51	91.20±0.36
**Mean**	14.20	13.70	14.33	13.91	212.90	199.17	223.27	214.03
**SD**	1.52	1.73	1.92	1.76	62.53	53.96	65.90	60.16

Values are expressed as mean ± standard deviation (n = 30).
